# Proposed mechanism of HCP → FCC phase transition in titianium through first principles calculation and experiments

**DOI:** 10.1038/s41598-018-20257-9

**Published:** 2018-01-31

**Authors:** Jia Xi Yang, Heng Lv Zhao, Hao Ran Gong, Min Song, Qing Qiang Ren

**Affiliations:** 1 State Key Laboratory of Powder Metallurgy, Central South University, Changsha, Hunan 410083 China; 2 Department of Materials Science and Engineering, Northwestern University, Evanston, IL 60201 USA

## Abstract

By means of first principles calculation and experiments, a detailed mechanism is proposed to include the stages of slip, adjustment, and expansion for the HCP → FCC phase transformation with the prismatic relation of ${{\{}{10}\bar{{1}}{0}{\}}}_{{hcp}}{\parallel }{\{}{1}\bar{{1}}{0}{{\}}}_{{fcc}}$ and ${{[}{0001}{]}}_{{hcp}}{\parallel }{[}{001}{{]}}_{{fcc}}$ in titanium. It is revealed that the formation of four FCC layers is preferable after the slip of Shockley partial dislocations of 1/6 $\langle {1}\bar{{2}}{10}\rangle$ on ${\{}{10}\bar{{1}}{0}{\}}$ planes, and that the adjustment of interplanar spacing and the volume expansion are energetically favorable and could happen spontaneously without any energy barrier. It is also found that the transformed FCC lattice first follows the *c*/*a* ratio (1.583) of HCP and then becomes an ideal FCC stru*c*ture (*c*/*a* = √2). The proposed mechanism could not only provide a deep understanding to the process of HCP → FCC prismatic transformation in titanium, but also clarify the controversy regarding volume expansion of HCP-FCC phase transition of titanium in the literature.

## Introduction

The phase transition between hexagonal-close-packed (HCP) and face-centered-cubic (FCC) structures has been well-regarded as one of the most important solid-solid phase transitions, and has raised great research interests during the past decades^1–26^. The HCP-FCC phase transition could be induced by mechanical deformation or temperature change^5–23^, and has been extensively observed in a lot of metals and alloys such as Ti^5–8^, Hf^9^, Zr^10^, Co^11–13^, Si^4^, Ta^14^, Al^15^, Au^16^, LnH^17^, MgTi^18^, TiAl^19^, TiZr^20^, CoCrMo^21^, FeMn^22^, FeMnSi^23^ etc. Especially, it is reported that the HCP-FCC phase transition could bring about the shape memory effect of several alloys^19,25^, and the appearance of the FCC structure can improve the ductility of the metals and alloys with the HCP structure^20,25^.

Since the discovery of Burgers in 1930s, it has been generally believed that during the HCP-FCC phase transition, the two structures should follow the orientation relation of ${{\{}{0001}{\}}}_{{hcp}}{\parallel }{\{}{111}{{\}}}_{{fcc}}$ and ${{[}{11}\bar{{2}}{0}{]}}_{{hcp}}{\parallel }{[}{110}{{]}}_{{fcc}}$ through the gliding of the Shockley partial dislocations on every two close-packed planes of HCP^26^. Very recently, however, a quite different orientation relation of ${{\{}{10}\bar{{1}}{0}{\}}}_{{hcp}}\parallel {{\{}{1}\bar{{1}}{0}{\}}}_{{fcc}}$ and ${{[}{0001}{]}}_{{hcp}}{\parallel }{[}{001}{{]}}_{{fcc}}$ is observed, for the first time, in pure Ti bulk under cryogenic plain-strain compression^6^, and such an orientation relationship can be named as prismatic relation. In addition, this prismatic relation between HCP and FCC structures is found in Ti single-crystal nanopillars under *[0001]* orientation tension by molecular dynamics simulation^7^, and is also discovered in polycrystalline Ti after rolling at room temperature^8^.

Regarding the prismatic relation of ${{\{}{10}\bar{{1}}{0}{\}}}_{{hcp}}\parallel {{\{}{1}\bar{{1}}{0}{\}}}_{{fcc}}$ and ${{[}{0001}{]}}_{{hcp}}{\parallel }{[}{001}{{]}}_{{fcc}}$, there are two mechanisms of HCP-FCC phase transformation so far in the literature^6–8,11^. Proposed by Hong *et al*. and confirmed later by Ren *et al*.^6,7^, the first mechanism states that the FCC structure is converted from the HCP structure by a series of Shockley partial dislocations with a Burgers vector of 1/6 $\langle {1}\bar{{2}}{10}\rangle$ on ${\{}{10}\bar{{1}}{0}{\}}$ stacking^6,11^. On the other hand, however, Wu *et al*. hold another view that the HCP → FCC phase transformation of the prismatic relation should be accomplished by nucleation via pure-shuffle and growth via shear-shuffle mechanism^8^. It should be pointed out that the detailed process of transformation is lacked in the first mechanism, and that the driving force to make atoms move a displacement along different orientations in small regions seems implausible in the second mechanism.

Furthermore, a controversy has appeared regarding volume expansion during HCP-FCC prismatic transformation in the first mechanism^6,8^. Hong *et al*. discovered the lattice expansion of 19.5% normal to the phase boundary in pure Ti bulk under cryogenic plain-strain compression after the HCP → FCC prismatic transition^6^, and Wu *et al*. also found the volume expansion of 14.2% in polycrystalline Ti through rolling at room temperature after the HCP → FCC prismatic transition^8^. Nevertheless, Wu *et al*. argued that, according to the first transformation mechanism^8^, the gliding of Shockley partial dislocations cannot generate the lattice expansion normal to the phase boundary^8^. It is, therefore, of vital importance to theoretically clarify such a controversy regarding volume expansion during the HCP-FCC prismatic transformation of titanium.

By means of first principles calculations and experiments, the present study is dedicated to propose a detailed mechanism of the HCP-FCC phase transformation with the prismatic relation of ${{\{}{10}\bar{{1}}{0}{\}}}_{{hcp}}\parallel {{\{}{1}\bar{{1}}{0}{\}}}_{{fcc}}$ and ${{[}{0001}{]}}_{{hcp}}{\parallel }{[}{001}{{]}}_{{fcc}}$. The metal of titanium (Ti) is intentionally selected as a typical example in the present study to show the prismatic transformation, as the HCP-FCC phase transformation of Ti has been extensively investigated in the literature^3,5–8,27–31^. The process and the energetics of the stages of slip, adjustment, and expansion are revealed and discussed to provide a deep understanding of the transformation. In addition, two HCP/FCC interface models are constructed and compared with each other to express the transformed FCC lattices, and the proposed mechanism of HCP-FCC transformation of Ti would be probably generalized to other systems as well.

## Methods

### Experiments

The materials used in this study are commercially pure Ti (99.999 at.%), and such a low level of impurity was found to have a negligible effect on HCP-FCC phase transition^32^. The as-received materials show an equiaxed grain structure with the grain size ranging from 20 to 30 μm. Small bars with a dimension of 20 × 10 × 1 mm were cut from the as-received plates, and then were cold-rolled at room temperature for multiple times with a thickness reduction of 0.5 mm per pass to reach a thickness reduction of 50%.

The TEM specimens were prepared using a double-jet electrolytic polisher (Struers TenuPol-5) and a solution of 60% methanol +35% butanol +5% per chloric acid at −30 °C with an applied voltage of 30 V. TEM and high-resolution TEM observations were performed using a FEI Titan G2 60–300 Cs-corrected microscope operated at 300 kV.

### Calculations

To find out the energy change and intrinsic mechanism of the HCP-FCC phase transition in Ti during the above experiments, the present first principles calculations are performed by means of Vienna ab initio simulation package (VASP)^33^ with the plane wave basis and projector-augmented wave (PAW) method^34^. The exchange and correlations items are described by generalized gradient approximation (GGA) of Perdew *et al*.^35^, and the cutoff energies are 500 eV for plane-wave basis. For each calculation, periodic boundary conditions are added in three directions and the energy criteria are 0.01 and 0.001 meV for relaxation and static calculations, respectively. For *k* space integration, the temperature smearing method of Methfessel–Paxton is used for dynamical calculation and the modified tetrahedron method of Blöchl-Jepsen-Andersen is chosen for static calculation^36^.

The lattice parameters of HCP Ti are first optimized as follows: *a* = 2.933 Å and *c*/*a* = 1.583, which agree well with corresponding experimental values^37^. To simulate the HCP-FCC prismatic phase transition, a unit cell of 14√3*a* × *a* × 1.583*a* is selected in the present study, and the *x*, *y*, and *z* axes of the unit cell are set as the $\langle {10}\bar{{1}}{0}\rangle$, $\langle {1}\bar{{2}}{10}\rangle$, and $\langle {0001}\rangle$ directions, respectively. It should be noted that there are 56 layers of prismatic ${\{}{10}\bar{{1}}{0}{\}}$ planes normal to the *x* axis, and such a number of layers has been tested to be enough to derive the detailed process of HCP-FCC phase transition. After the test calculations, the *k*-meshes of 1 × 11 × 7 and 1 × 13 × 9 are adopted for relaxation and static calculations, respectively. During each step of the phase transformation, the unit cell is optimized until the force acting on each atom reaches 0.001 eV/Å. It should be pointed out that the above bulk model, instead of the surface model with vacuum layer^38^, is purposely selected in the present study, since the HCP-FCC phase transition has been mainly observed inside the sample, rather than located at the surface area^6,8^.

## Results

During the prismatic transformation with the orientation relation of ${\langle {0001}\rangle }_{{hcp}}{\parallel }{\langle {001}\rangle }_{{fcc}}$, and ${{\{}{10}\bar{{1}}{0}{\}}}_{{hcp}}\parallel {{\{}{1}\bar{{1}}{0}{\}}}_{{fcc}}$, the stacking of -A-B-C-D- in the ${\{}{10}\bar{{1}}{0}{\}}$ planes of the HCP structure would transform to the stacking of -A-B-A-B- in the ${\{}1\bar{{1}}0{\}}$ plane of the FCC structure. A detailed mechanism of the HCP-FCC prismatic transformation is thus proposed in the present study to include the stages of slip, adjustment, and expansion. The energy change during each stage of the transformation will be calculated and compared with each other, in order to get a deep understanding of the transformation mechanism. It is revealed that the FCC structure is transformed from the HCP structure by the slip of Shockley partial dislocations of 1/6 $\langle {1}\bar{{2}}{10}\rangle$ on every four layers of ${\{}{10}\bar{{1}}{0}{\}}$ planes. Moreover, the derived results could clarify the above-mentioned controversy regarding volume expansion during the HCP-FCC prismatic transformation^6–8^.

### Slip and adjustment

**Figure 1:**
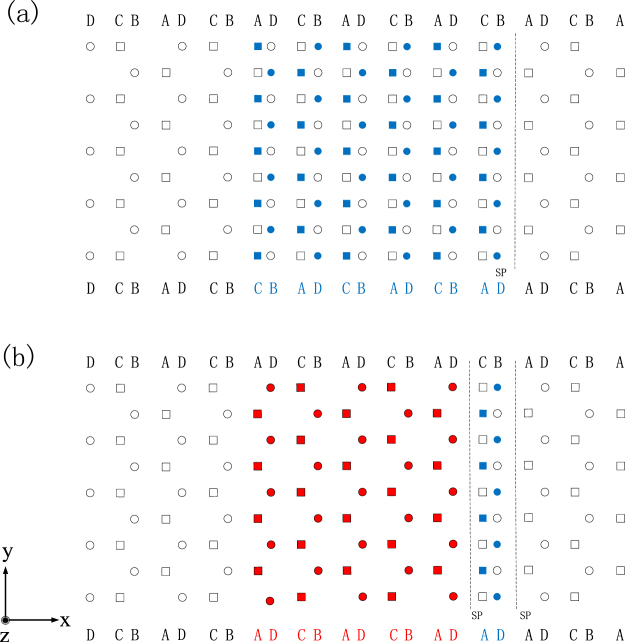
Atomic positions of HCP Ti after (**a**) the first and (**b**) the second slips of Shockley partial dislocation of 1/6 $< {1}\bar{{2}}{10} >$ on $\{{10}\bar{{1}}{0}\}$ planes. The *x* axis is along $< {10}\bar{{1}}{0}{ > }_{{\rm{hcp}}}$ and $< {1}\bar{{1}}{0}{ > }_{{\rm{fcc}}}$, the *y* axis is along $< {1}\bar{{2}}{10}{ > }_{{\rm{hcp}}}$ and $< {110}{ > }_{{\rm{fcc}}}$, and the *z* axis is along $< {0001}{ > }_{{\rm{hcp}}}$ and $< {001}{ > }_{{\rm{fcc}}}$. The hollow square and circular symbols represent atoms on $\{{0001}\}$ and $\{{0002}\}$ planes, respectively. The filled blue and red symbols are atoms after the first and second slips of Shockley partial dislocation, respectively, and SP stands for the slip plane.

To express the slip process clearly during the prismatic transformation, Fig. 1 displays atomic positions of HCP Ti after the first and second slips of Shockley partial dislocation of 1/6 $\langle {1}\bar{{2}}{10}\rangle$ on ${\{}{10}\bar{{1}}{0}{\}}$ planes. The interplanar spacing (√3/3*a*) of ${\{}{10}\bar{{1}}{0}{\}}$ between *A* and *B* or *C* and *D*, as shown in Fig. 1, is twice as much as that (√3/6*a*) between *D* and *A* or *B* and *C*, suggesting that the slip plane should be located just between *A* and *B* or *C* and *D*. It should be noted that the slip of a Shockley partial dislocation must have a start plane and a terminal plane due to the limited affected range of the partial dislocation. After the first slip of the Shockley partial dislocation of 1/6 $\langle {1}\bar{{2}}{10}\rangle$ displayed in Fig. 1(a), the hollow symbols within the affected range would move to the filled symbols, indicating that the HCP stacking of D-A-B-C close to the slip plane (SP) has been changed to the stacking of D-A-D-A for the ${\{}{1}\bar{{1}}{0}{\}}$ plane of FCC. Similarly, the second slip of the Shockley partial dislocation of 1/6 $\langle {1}\bar{{2}}{10}\rangle$ in Fig. 1(b) could harvest another two D-A stacking of FCC ${\{}{1}\bar{{1}}{0}{\}}$ plane from the B-C stacking of HCP $\{{10}\bar{{1}}{0}\}$ planes. That is to say, two ${\{}{1}\bar{{1}}{0}{\}}$ planes of FCC are therefore formed by each slip of the Shockley partial dislocation of 1/6 $\langle {1}\bar{{2}}{10}\rangle$.

It is of importance to find out the energy changes during a series of slips of the Shockley partial dislocation of 1/6 $\langle {1}\bar{{2}}{10}\rangle$. To calculate the energy barrier, six points are chosen along the slip path of the Shockley partial dislocation with equal spacing, and 26 layers are set as the affected region for the first slip of the partial dislocation. The atoms in the phase transition region (affected region) are only allowed to relax along the direction of *x*-axis perpendicular to the prismatic plane of HCP $\{{10}\bar{{1}}{0}\}$, and are fixed along other directions, while the atoms outside the phase transition region are all kept fixed. Such a setting of optimization is similar to others in the literature^39–41^ and seems reasonable to obtain the energies of various intermediate structures during the slip process. The energy barrier is derived as the difference between total energies of the saddle point and initial image, and the relative energy is defined as the energy difference between the initial and final images.

**Figure 2:**
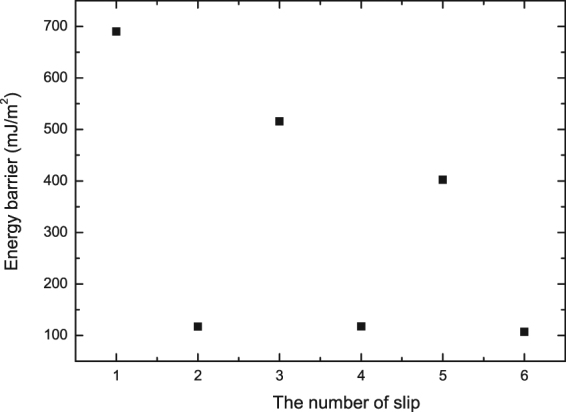
Energy barriers during various slips of Shockley partial dislocation of 1/6 $< {1}\bar{{2}}{10} >$ on Ti $\{{10}\bar{{1}}{0}\}$ planes.

After a series of calculations, Fig. 2 summarizes the derived energy barriers during six slips of Shockley partial dislocation of 1/6 $\langle {1}\bar{{2}}{10}\rangle$ on $\{{10}\bar{{1}}{0}\}$ planes of Ti. It can be seen obviously from this figure that the energy barriers of the first, third, and fifth slips of Shockley partial dislocations are 690, 520, and 410 mJ/m^2^, respectively, which are much bigger than the corresponding values of 117, 118, and 106 mJ/m^2^ for the second, fourth, and sixth slips. Such a dramatic comparison suggests that the second, fourth, and sixth slips of Shockley partial dislocations should be energetically much easier than the precedent slip (first, third, and fifth), and could be triggered spontaneously if the energy barrier of the precedent slip has been overcome. In other words, two slips of Shockley partial dislocations of 1/6 $\langle {1}\bar{{2}}{10}\rangle$ on $\{{10}\bar{{1}}{0}\}$ planes should happen together from the point of view of energetics. Considering that each slip can generate two $\{{1}\bar{{1}}{0}\}$ planes of FCC as related before, it therefore follows that the formation of four $\{{1}\bar{{1}}{0}\}$ planes of FCC should be preferred during the process of HCP-FCC prismatic transformation, and such a transition mode of four FCC layers is observed for the first time during the HCP-FCC phase transition.

We now turn to investigate the adjustment during the process of prismatic HCP-FCC transformation. As shown in Fig. 1 and related before, the interplanar spacing of HCP $\{{10}\bar{{1}}{0}\}$ between D and A is quite different from that between A and B, while the interplanar spacing of FCC $\{{1}\bar{{1}}{0}\}$ planes should be the same. It therefore follows that the interplanar spacing of the transformed FCC $\{{1}\bar{{1}}{0}\}$ planes just after slip should be adjusted, i.e., the uneven interplanar spacing of HCP $\{{10}\bar{{1}}{0}\}$ is adjusted to become even in FCC $\{{1}\bar{{1}}{0}\}$ planes. As a typical example, after six slips of Shockley partial dislocation of 1/6 $\langle {1}\bar{{2}}{10}\rangle$, the relative energy between FCC $\{{1}\bar{{1}}{0}\}$ planes without and with the adjustment of the interplanar spacing is calculated to be −102 meV/atom, indicating that the adjustment of interplanar spacing is energetically favorable and could happen spontaneously without any energy barrier. It should be pointed out slip and adjustment should happen simultaneously during the actual HCP-FCC prismatic transformation, and the above separation of slip and adjustment is just for the sake of expression.

**Figure 3:**
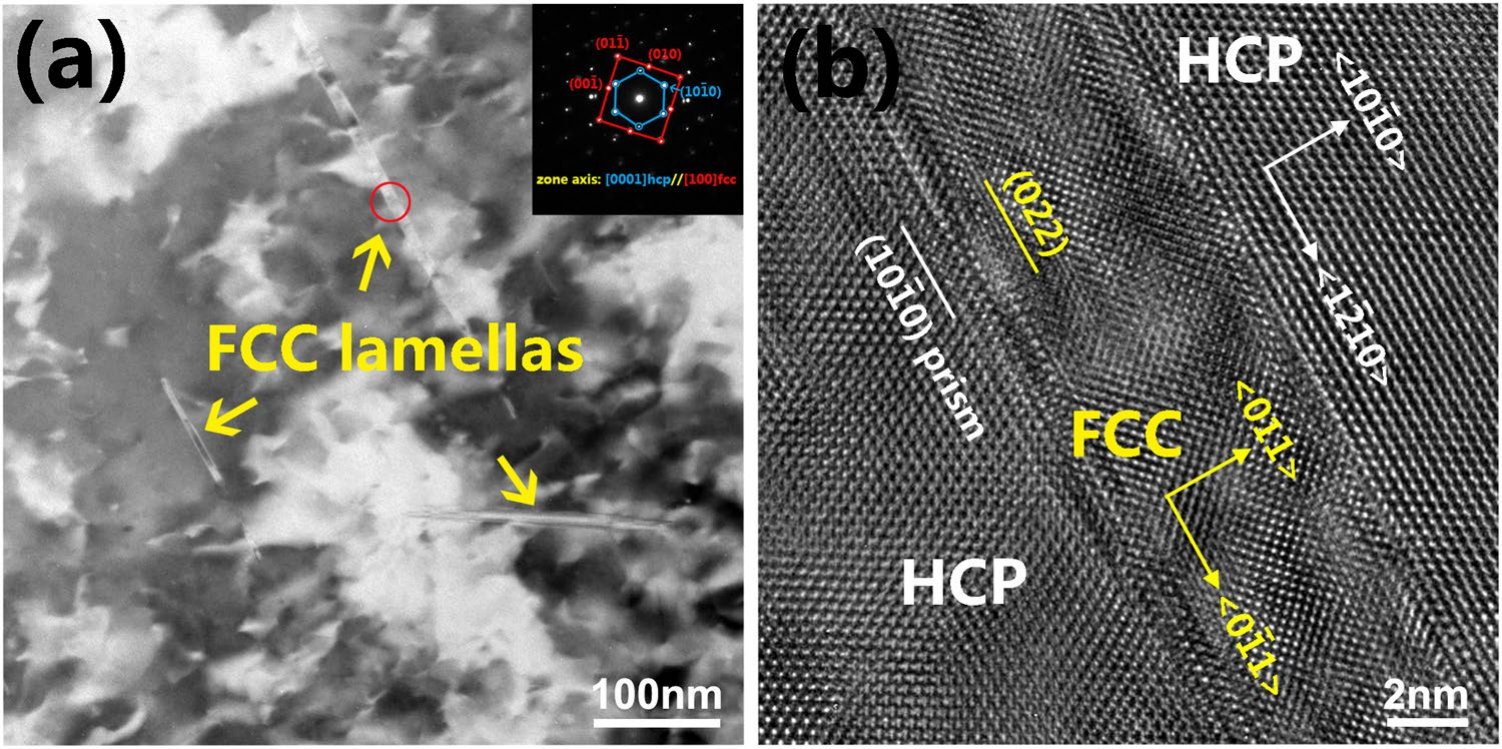
(**a**) A TEM image and the corresponding SAED pattern of the cold-rolled Ti with a thickness reduction of 50%. (**b**) An HRTEM image of the red circle area in (**a**), showing the orientation relation of the HCP-FCC interface.

It is of interest to compare the above predicted results with experiments. Accordingly, Fig. 3 shows the TEM and HRTEM images of the cold-rolled Ti with a thickness reduction of 50%. One can see clearly from Fig. 3(a) that several lamellar phases have appeared in the HCP matrix and the lamellas are identified to be the FCC structure of Ti. As shown in Fig. 3(b), the orientation relationship between HCP and FCC is indexed as ${\langle {0001}\rangle }_{{hcp}}{\parallel }{\langle {100}\rangle }_{{fcc}}$ and ${{\{}{10}\bar{{1}}{0}{\}}}_{{hcp}}\parallel {{\{}{011}{\}}}_{{fcc}}$, which matches well with that in Fig. 1. In addition, it could be discerned from Fig. 3(b) that the interplanar spacing of FCC *{011}* planes has been indeed adjusted to become even. These nice agreements between calculation and experiments suggest that the proposed transition mode of FCC layers by means of slip and adjustment should be realistic to express the HCP-FCC prismatic transformation.

### Expansion

After the slip and adjustment, the nearest-neighbor distances in the $< {1}\bar{{1}}{0} >$ and $< {110} >$ directions of the transformed FCC region are named d1 and d2 with the values of √3/2*a* and 1*a*, respectively. The ratio of d1/d2 in the transformed FCC is calculated to be √3/2, which seems not equal to the corresponding value of 1 for an ideal FCC structure. It is, therefore, of importance to investigate whether or not the transformed FCC phase has a tendency to become the ideal FCC lattice through the change of the volume.

**Figure 4:**
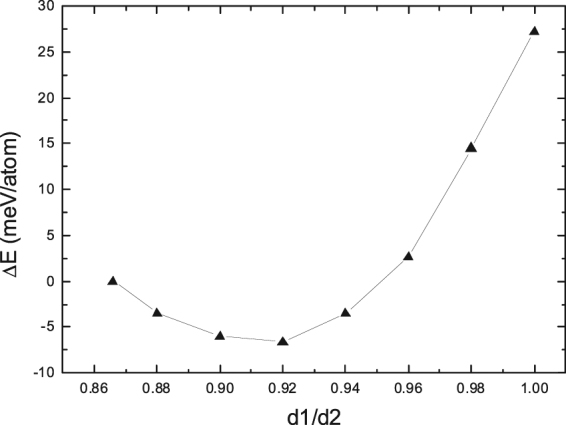
Energy difference (ΔE) of the unit cell as a function of d1/d2 with respect to the total energy of the unit cell just after the slip and adjustment (d1/d2 = √3/2). d1 and d2 are the nearest-neighbor distances in the $< {1}\bar{{1}}{0} >$ and $< {110} >$ directions, respectively.

In the present study, the volume of the transformed FCC phase is optimized through the variation of the d1/d2 ratio. The range of the d1/d2 ratio is varied from the initial state just after the slip and adjustment (d1/d2 = √3/2) to the ideal FCC lattice (d1/d2 = 1.0) with an interval of 0.02. The total energy of the unit cell at each ratio of d1/d2 is calculated, respectively, and Fig. 4 shows the obtained energy difference of the unit cell as a function of d1/d2 with respect to the total energy of the unit cell just after the slip and adjustment (d1/d2 = √3/2).

One can discern from Fig. 4 that with the increase of the d1/d2 ratio, the total energy of the unit cell first decreases and then increase, with a minimum of energy curve at the d1/d2 ratio of 0.92, which corresponds to the optimized volume of the unit cell. Apparently, the optimized d1/d2 ratio of 0.92 is bigger than the initial value of √3/2 just after the slip and adjustment, suggesting that the volume of the unit cell has indeed expanded. Such a volume expansion during the HCP → FCC prismatic transformation of Ti revealed from the present study matches well with similar experimental observations in the literature^6,8^.

We now investigate the energy change during volume expansion. As shown in Fig. 4, a gradual decrease of total energy appears until the volume expands at the critical point (d1/d2 = 0.92), and the relative energy between the initial point (√3/2) and the critical point (d1/d2 = 0.92) is derived to be −7 meV/atom. These features imply that the volume expansion during the HCP → FCC prismatic transformation should be thermodynamically favorable and could take place spontaneously without any energy barrier.

As related before, there is a controversy regarding volume expansion during the HCP → FCC prismatic transformation in the literature, i.e., whether or not the gliding of Shockley partial dislocations can cause the lattice expansion normal to the phase boundary^6,8^. In the present study, the volume expansion perpendicular to the phase boundary after the slip of Shockley partial dislocations has been indeed observed during the HCP → FCC prismatic transformation of Ti, and this volume expansion is energetically favorable and could take place spontaneously without any energy barrier. All the above statements revealed from the present study not only provide a deep understanding of the process of HCP-FCC prismatic transformation, but also clarify the controversy regarding volume expansion during the prismatic transition in the literature^6,8^.

## Discussion

We have so far proposed a detailed mechanism of the HCP → FCC prismatic transformation with the stages of slip, adjustment, and expansion. It is revealed that an energy barrier of more than 600 mJ/m^2^ should be overcome during the slips of Shockley partial dislocations of 1/6 $< {1}\bar{{2}}{10} >$ on $\{{10}\bar{{1}}{0}\}$ planes, while the adjustment and expansion during the HCP → FCC prismatic transformation are energetically favorable with negative relative energies and could take place spontaneously without any energy barrier. This comparison suggests that slip should be the predominant stage during the HCP → FCC prismatic transformation. In other words, once the slip stage is triggered, the adjustment and expansion would occur spontaneously, and the HCP → FCC prismatic transformation could be called slip-controlled phase transition from the point of view of energetics.

During the process of the HCP → FCC prismatic transformation as shown in Fig. 1, it should be noted that the length of the *y* ($< {110} >$) or *z* ($< {001} >$) axis of the FCC lattice is the same as that of HCP, while the *x* axis ($< {1}\bar{{1}}{0} >$) of the FCC lattice is allowed to relax. The *z*/*y* ratio of the transformed FCC structure keeps the optimized HCP value of 1.583 as related before, and such a ratio is quite different from the corresponding value of √2 for an ideal FCC structure. It is, therefore, of interest to further compare the above transformed FCC lattice with an ideal FCC structure (*z*/*y* = √2).

**Figure 5:**
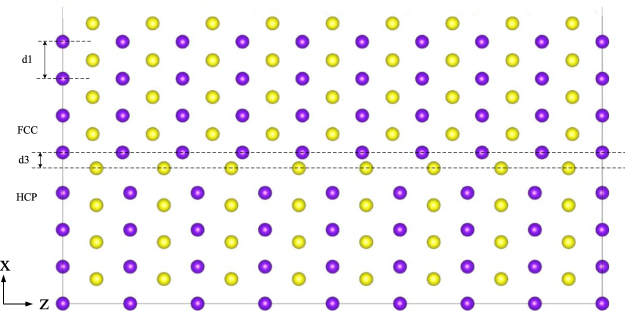
Schematic illustrations of the HCP/FCC interface model of I2 with the unit cell of 4√3*a* × *a* × (8 × 1.583*a*) before volume expansion. The *x* axis is along $< {10}\bar{{1}}{0}{ > }_{{\rm{hcp}}}$ and $< {1}\bar{{1}}{0}{ > }_{{\rm{fcc}}}$, the *y* axis is along $< {1}\bar{{2}}{10}{ > }_{{\rm{hcp}}}$ and $< {110}{ > }_{{\rm{fcc}}}$, and the *z* axis is along $< {0001}{ > }_{{\rm{hcp}}}$ and $< {001}{ > }_{{\rm{fcc}}}$. The purple and yellow balls represent atoms on $\{{0001}\}$ and $\{{0002}\}$ planes, respectively. d1 and d2 are the nearest-neighbor distance in the $< {1}\bar{{1}}{0} >$ and $< {110} >$ directions, respectively. d3 is the interface spacing between HCP and FCC.

A new HCP/FCC interface model is thus constructed as follows: the length of the *y* ($< {110} >$) axis of the FCC lattice is the same as that of HCP, the *z* ($< {001} >$) axis of FCC is set with the *z*/*y* ratio of √2, and the *x* axis ($< {1}\bar{{1}}{0} >$) of FCC lattice is allowed to relax. Specifically, a unit cell of 4√3*a* × *a* × (8 × 1.583*a*) is chosen for the HCP/FCC interface model, which is shown schematically in Fig. 5. It should be noted that in the z direction, nine layers of FCC atoms are intentionally put to match eight layers of HCP atoms, in order to attain the *z*/*y* ratio of FCC very close to √2. For simplicity, this new constructed HCP/FCC interface model is called I2, and the original one in Fig. 1 after the process of slip, adjustment, and expansion is named I1.

**Figure 6:**
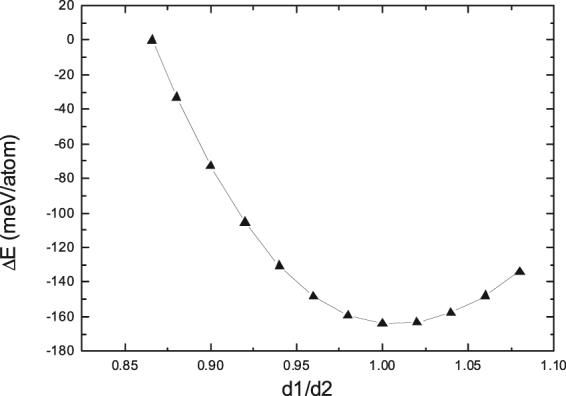
Energy difference (ΔE) of the HCP/FCC interface model of I2 as a function of d1/d2 with respect to the total energy of I2 before volume expansion (d1/d2 = √3/2). d1 and d2 are the nearest-neighbor distance in the $< {1}\bar{{1}}{0} >$ and $< {110} >$ directions, respectively.

To find out the optimized structure of I2, the d1/d2 ratio is varied within the range from√3/2 to 1.08 with an interval of 0.02. The total energy of the I2 model is calculated as a function of d1/d2, and Fig. 6 displays the derived energy difference of the I2 model with respect the total energy of I2 before volume expansion (d1/d2 = √3/2). Interestingly, the total energy of I2 is minimized when the ratio of d1/d2 increases to the critical point of 1.0. That is to say, the volume of the optimized I2 structure has expanded dramatically, and the FCC part of the optimized I2 structure is very close to an ideal FCC structure.

It is of importance to compare the thermodynamic stability of the interface models of I1 and I2. Accordingly, the interface energy is calculated according to the following form^42^:

1 $${E}_{{int}}=\frac{{E}_{{total}}-{{\rm{E}}}_{{HCP}}-{{E}}_{{FCC}}}{{A}},$$

where *E*_total_, *E*_HCP_, and *E*_FCC_ are total energies of the interface, corresponding HCP and FCC bulks, respectively, and *A* is the surface area of the interface. After the calculation, the obtained interface energy of I1 is 0.33 J/m^2^, which seems much smaller than the corresponding value of 1.80 J/m^2^ for I2. This big difference of interface energy implies that the HCP/FCC interface model of I1 should be energetically more favorable than I2, and is more likely to be formed during the HCP → FCC prismatic transformation.

Furthermore, the total energies of the FCC lattices in the HCP/FCC interface models of I1 and I2 are also calculated and compared with each other. In other words, after the removal of the HCP part in the interface models of I1 and I2, the lattice parameters and atomic positions of the FCC lattice are kept, in order to obtain its total energy. Consequently, the total energies of the FCC lattices in I1 and I2 are derived to be −7.645 and −7.712 eV/atom, respectively. The lower energy of FCC in I2 suggests that the FCC bulk in I2 should be thermodynamically more stable than that in I1.

From the above comparisons of interface energy and bulk energies of FCC, a probably useful conclusion could be drawn for the HCP → FCC prismatic transformation. During the initial stage of the HCP → FCC prismatic transition, the transformed FCC lattice would energetically prefer a *c*/*a* ratio of 1.583 due to the smaller interface energy of HCP/FCC interface (I1). Subsequently, however, the transformed FCC lattice with the *c*/*a* ratio of 1.583 would have a tendency to become the ideal FCC lattice with the *c*/*a* ratio of √2 (I2), in order to lower its total energy.

**Figure 7:**
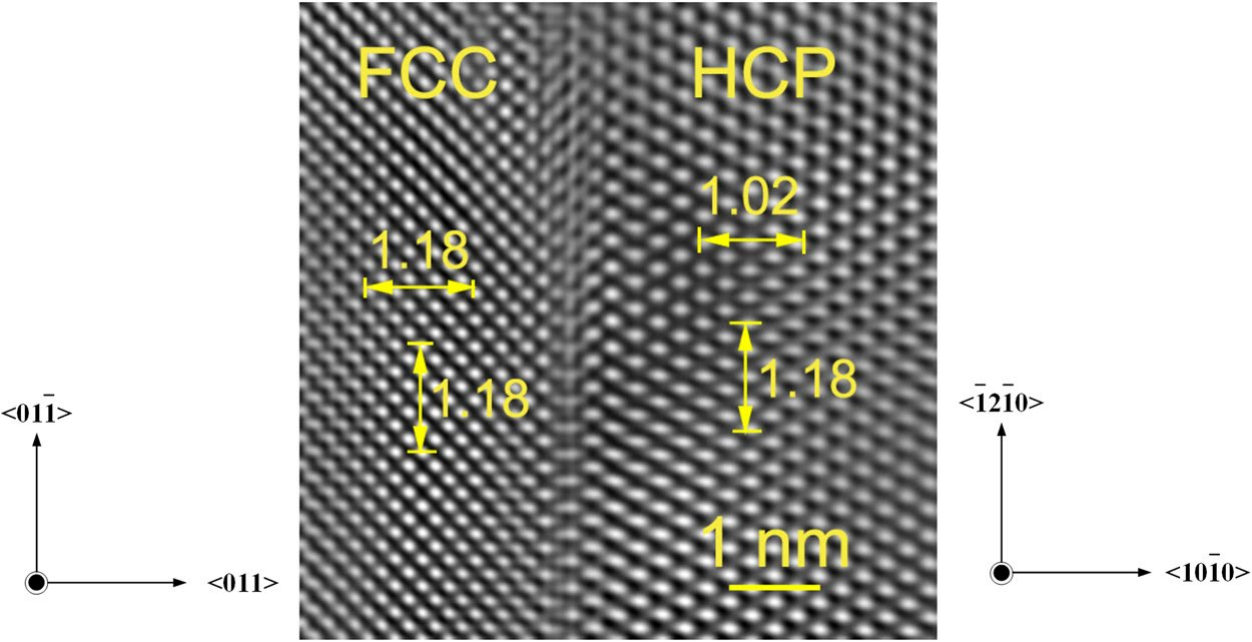
Fourier-filtered HRTEM image of ${\{{011}\}}_{{\rm{fcc}}}\parallel {\{{10}\bar{{1}}{0}\}}_{{\rm{hcp}}}$ interface area in Fig. 3(b).

In addition, Fig. 7 shows the Fourier-filtered HRTEM image of ${\{{011}\}}_{{\rm{fcc}}}$//${\{{10}\bar{{1}}{0}\}}_{{\rm{hcp}}}$ interface area in Fig. 3(b), and the measured interplanar spacing of eight planes of HCP and FCC Ti. Several characteristics could be deduced from this figure. Firstly, the interplanar spacing (1.18 nm) of eight ${\{{1}\bar{{2}}{10}\}}_{{\rm{hcp}}}$ planes is the same as that of eight ${\{{0}\bar{{1}}{1}\}}_{{\rm{fcc}}}$ planes. This experimental observation from the present study indicates that the length of the *y* ($< {0}\bar{{1}}{1} >$) axis of the FCC lattice should be the same as that ($< {1}\bar{{2}}{10}{ > }_{{\rm{hcp}}}$) of HCP, which confirms the above theoretical prediction regarding the constant length of *y* axis in the I1 and I2 interfaces models during HCP-FCC prismatic transformation.

Secondly, the interplanar spacing (1.18 nm) in the ${\{{0}\bar{{1}}{1}\}}_{{\rm{fcc}}}$ direction is also identical to that in the ${\{{011}\}}_{{\rm{fcc}}}$ direction. In other words, the d1/d2 ratio of the obtained FCC lattice in Fig. 7 is the critical point of 1.0 for an ideal FCC structure, which agrees well with the theoretically predicted value of d1/d2 ratio as shown in Fig. 6. Such an experimental measurement suggests that the transformed FCC lattice in Fig. 7 could be probably very similar to the FCC part of the I2 interface model.

Thirdly, the experimental lattice expansion in the *x* direction (${ < {10}\bar{{1}}{0} > }_{{\rm{hcp}}}$) due to the HCP-FCC transition is calculated, from the interplanar spacing in Fig. 7, to be 15.7%, which matches well with the corresponding theoretical value of 15.4% derived from Fig. 6. It should be noted that the above data of lattice expansion normal to the HCP/FCC interface from the present study is consistent with the corresponding values of 14.2%^8^ and 19.5%^6^ estimated from other experiments. All the above good agreements between theoretical and experimental observations regarding *y* axis, d1/d2 ratio, and lattice expansion indicate that the proposed transition mechanism by means of I1 and I2 interface models in the present study should be relevant to reflect the intrinsic characteristics of HCP-FCC prismatic transformation.

Finally, we would like to discuss a little bit about the differences between the present mechanism and the two already in the literature^6–8^. In the present study, a detailed mechanism has been proposed for the HCP → FCC phase transformation to include the stages of slip, adjustment, and expansion; the formation of four FCC layers is preferred during the slip process; the stages of adjustment and volume expansion could happen spontaneously after the slip without any energy barrier; the transformed FCC lattice would first follow the *c*/*a* ratio (1.583) of HCP and then become an ideal FCC structure (*c*/*a* = √2); the present mechanism could clarify the controversy regarding volume expansion of HCP-FCC phase transition in the two mechanisms as related before^6–8^. All the above points from the present study have been observed for the first time and are quite different from the discoveries in the mechanisms by Hong *et al*. and Ren *et al*. in the literature^6–8^. It should be noted that the present mechanism is observed from the HCP metal of Ti and would probably be generalized to the HCP-FCC phase transition in other systems. Experimental and theoretical studies are welcome to confirm the predictions in the present study, and to propose other possible mechanism for the HCP-FCC phase transition.

## Conclusions

Based on first principles calculations and experiments, the present study has proposed that the HCP → FCC phase transformation in titanium with the prismatic relation of ${\{{10}\bar{{1}}{0}\}}_{{\rm{hcp}}}\parallel {\{{1}\bar{{1}}{0}\}}_{{\rm{fcc}}}$ and ${[{0001}]}_{{\rm{hcp}}}\parallel {[{001}]}_{{\rm{fcc}}}$ should follow the stages of slip, adjustment, and expansion. The slip of Shockley partial dislocations of 1/6 $< {1}\bar{{2}}{10} >$ on $\{{10}\bar{{1}}{0}\}$ planes should overcome an energy barrier of more than 600 mJ/m^2^, and the formation of four FCC layers is energetically preferable as a result of the slip. On the other hand, the adjustment of interplanar spacing and the volume expansion could happen spontaneously with negative relative energies, which could clarify the controversy regarding volume expansion during the HCP → FCC prismatic transition in the literature. In addition, two HCP/FCC interface models are constructed and compared with each other, indicating that the FCC lattice energetically prefers the *c*/*a* ratio (1.583) of HCP at the initial stage, and then has a tendency to become the ideal FCC structure (*c*/*a* = √2). The predicted results are in good agreement with corresponding experimental observations, and the proposed mechanism of the HCP → FCC prismatic transition of titanium would be probably generalized to other metals and alloys as well.

## References

[1] Hennig RG (2005). Impurities block the α to ω martensitic transformation in titanium. Nat. Mater..

[2] Trinkle DR (2003). New mechanism for the α to ω martensitic transformation in pure titanium. Phys. Rev. Lett..

[3] Jankowski AF, Wall MA (1994). Formation of face-centered cubic titanium on a Ni single crystal and in Ni/Ti multilayers. J. Mater. Res..

[4] Duclos SJ, Vohra YK, Ruoff A (1987). L. hcp-to-fcc transition in Silicon at 78 GPa and studies to 100 GPa. Phys. Rev. Lett..

[5] Manna I, Chattopadhyay PP, Nandi P, Banhart F, Fecht HJ (2003). Formation of face-centered-cubic titanium by mechanical attrition. J. Appl. Phys..

[6] Hong DH, Lee TW, Lim SH, Kim WY, Hwang SK (2013). Stress-induced hexagonal close-packed to face-centered cubic phase transformation in commercial-purity titanium under cryogenic plane-strain compression. Scripta Mater..

[7] Ren JQ, Sun QY, Xiao L, Ding XD, Sun J (2014). Phase transformation behavior in titanium single-crystal nanopillars under [0001] orientation tension: A molecular dynamics simulation. J. Comp. Mater. Sci..

[8] Wu HC (2016). Rolling-induced face centered cubic Titanium in Hexagonal Close Packed Titanium at room temperature. Sci. Rep-UK.

[9] Ni S, Zheng XZ, Liao XZ, Song M (2014). Phases in pure hafnium. Phil. Mag. Lett..

[10] Manna I, Chattopadhyay PP, Banhart F, Fecht HJ (2002). Formation of face-centered-cubic zirconium by mechanical attrition. Appl. Phys. Lett..

[11] Waitz T, Karnthaler HP (1997). The fcc to hcp Martensitic phase transformation in CoNi studied by TEM and AFM methods. Acta Mater..

[12] Wu X (2005). Strain-induced grain refinement of cobalt during surface mechanical attrition treatment. Acta Mater..

[13] Edalati K, Toh S, Arita M, Watanabe M, Horita Z (2013). High-pressure torsion of pure cobalt: hcp-fcc phase transformations and twinning during severe plastic deformation. Appl. Phys. Lett..

[14] Janish MT, Kotula PG, Boyce BL, Carter CB (2015). Observations of fcc and hcp tantalum. J. Mater. Sci..

[15] Akahama Y, Nishimura M, Kinoshita K, Kawamura H (2006). Evidence of a fcc-hcp transition in aluminum at multimegabar pressure. Phys. Rev. Lett..

[16] Fan ZX (2014). Surface modification-induced phase transformation of hexagonal close-packed gold square sheets. Nat. Commun..

[17] Mintz MH, Hiershler D, Hadari Z (1976). Systematic study of the H.C.P. → F.C.C. transitions in the heavier LnH_2_-InH_3_ systems. J. Less Common Metals.

[18] Asano K, Enoki H, Akiba E (2009). Synthesis of HCP, FCC and BCC structure alloys in the Mg–Ti binary system by means of ball milling. J. Alloy. Compd..

[19] Banerjee R, Ahuja R, Fraser H (1996). Dimensionally Induced Structural transformations in Titanium-Aluminum multilayers. Phys. Rev. Lett..

[20] Zhang ZB (2014). Enhancement of TiZr ductility by hcp–fcc martensitic transformation after severe plastic deformation. Mat. Sci. Eng. A.

[21] Dahn NC, Morphy D, Rajan K (1984). Kinetics of the Martensitic F.C.C. → H.C.P. transformation in Co-Cr-Mo alloy powders. Acta Mater..

[22] Cotes SM, Guillermet AF, Sade M (2004). Fcc/Hcp Martensitic transformation in the Fe-Mn system: Part II. driving force and thermodynamics of the nucleation process. Metall. Mater. Trans. A.

[23] Sato A, Yamaji Y, Mori T (1986). Physical properties controlling shape memory effect in Fe-Mm-Si alloys. Acta Mater..

[24] Sun JW, Wang SL, Yan ZW, Peng HB, Wen YH (2015). Origin of shape memory effect in Co–Ni alloys undergoing fcc-hcp martensitic transformation. Mat. Sci. Eng. A.

[25] Wu X, Tao N, Hong Y, Lu J, Lu K (2005). γ → ε Martensite transformation and twinning deformation in fcc cobalt during surface mechanical attrition treatment. Scripta Mater..

[26] Burgers WG (1934). On the process of transition of the cubic-body-centered modification into the hexagonal-close-packed modification of zirconium. Physica.

[27] Heerden DV, Josell D, Shechtman D (1996). The formation of F.C.C. Titanium in Titanium-Aluminum multilayers. Acta Mater..

[28] Lai JB, Chen LJ, Liu CS (1999). Ion beam induced formation of metastable fcc-Ti phase in the epitaxial Ti/Cu/(111)Si structures. Micron.

[29] Franklin E, Wawner Jr, Lawless KR (1969). Epitaxial growth of Titanium thin films. J. Vac. Sci. Technol. B.

[30] Chakraborty J, Kumar K, Ranjan R, Chowdhury SG, Singh SR (2011). Thickness-dependent fcc-hcp phase transformation in polycrystalline titanium thin films. Acta Mater..

[31] Chatterjee P, Gupta SPS (2001). An X-ray diffraction study of strain localization and anisotropic dislocation contrast nanocrystalline titanium. Phil. Mag. Lett..

[32] Hennig RG, Lenosky TJ, Trinkle DR, Rudin SP, Wilkins JW (2008). Classical potential describes martensitic phase transformations between the α, β, and ω titanium phases. Phys. Rev. B.

[33] Kresse G, Hafner J (1993). Ab initio molecular dynamics for liquid metals. Phys. Rev. B.

[34] Kresse G, Joubert D (1999). From ultrasoft pseudopotentials to the projector augmented-wave method. Phys. Rev. B.

[35] Perdew JP (1992). Atoms, molecules, solids, and surfaces: Applications of the generalized gradient approximation for exchange and correlation. Phys. Rev. B.

[36] Blöchl PE (1994). Projector augmented-wave method. Phys. Rev. B.

[37] Vohra YK, Spencer PT (2001). Novel g-phase of Titanium metal at megabar pressures. Phys. Rev. Lett..

[38] Ogata S, Li J, Yip S (2005). Energy Landscape of Deformation Twinning in bcc and fcc Metals. Phys. Rev. B.

[39] Kwasniak P, Garbacz H, Kurzydlowski KJ (2016). Solid Solution Strengthening of Hexagonal Titanium Alloys: Restoring Forces and Stacking Faults Calculated From First Principles. Acta Mater..

[40] Yan JA, Wang CY, Wang SY (2004). Generalized-stacking-fault energy and dislocation properties in bcc Fe: A first-principles study. Phys. Rev. B.

[41] Wu X, Wang R, Wang S (2010). Generalized-stacking-fault energy and surface properties for HCP metals: A first-principles study. Appl. Surf. Sci..

[42] Lu S, Hu QM, Punkkinen MPJ, Johansson B, Vitos L (2013). First-Principles Study of fcc-Ag/bcc-Fe Interfaces. Phys. Rev. B.

